# Workshops without Walls: Broadening Access to Science around the World

**DOI:** 10.1371/journal.pbio.1001118

**Published:** 2011-08-02

**Authors:** Betül K. Arslan, Eric S. Boyd, Wendy W. Dolci, K. Estelle Dodson, Marco S. Boldt, Carl B. Pilcher

**Affiliations:** 1 NASA Astrobiology Institute Center for Ribosomal Origins and Evolution, Georgia Institute of Technology, Atlanta, Georgia, United States of America; 2 School of Biology, Georgia Institute of Technology, Atlanta, Georgia, United States of America; 3 NASA Astrobiology Institute Astrobiology Biogeocatalysis Research Center, Montana State University, Bozeman, Montana, United States of America; 4 Department of Chemistry and Biochemistry, Montana State University, Bozeman, Montana, United States of America; 5 NASA Astrobiology Institute, NASA Ames Research Center, Moffett Field, California, United States of America; 6 Lockheed Martin, NASA Ames Research Center, Moffett Field, California, United States of America

## Abstract

The National Aeronautics and Space Administration (NASA) Astrobiology Institute (NAI) conducted two “Workshops Without Walls” during 2010 that enabled global scientific exchange—with no travel required. The second of these was on the topic “Molecular Paleontology and Resurrection: Rewinding the Tape of Life.” Scientists from diverse disciplines and locations around the world were joined through an integrated suite of collaborative technologies to exchange information on the latest developments in this area of origin of life research. Through social media outlets and popular science blogs, participation in the workshop was broadened to include educators, science writers, and members of the general public. In total, over 560 people from 31 US states and 30 other nations were registered. Among the scientific disciplines represented were geochemistry, biochemistry, molecular biology and evolution, and microbial ecology. We present this workshop as a case study in how interdisciplinary collaborative research may be fostered, with substantial public engagement, without sustaining the deleterious environmental and economic impacts of travel.

## Introduction

The great astronomer and physicist Sir Isaac Newton (1642–1727) once remarked, “We build too many walls and not enough bridges.” Yet now, nearly 300 years later during an era of vastly improved technologies, scientists still struggle to overcome barriers in scientific communication. An essential goal of the National Aeronautics and Space Administration (NASA) Astrobiology Institute (NAI) [1] is to bring scientists together across distance, disciplines, and organizational boundaries to seek answers to the profound questions of astrobiology: How did life begin and evolve? Are we alone in the universe? And what is the future of life on earth and beyond? The NAI is using modern collaborative technologies to improve scientific communication and discourse among scientists—and between scientists and the public. Such lines of communication not only enable multidisciplinary science, but also provide a window for the public to see how science is conducted and experience undiluted scientific results. Open access to science is desperately needed at a time when public understanding in critical areas, such as evolution and global climate change, is of the utmost importance [2]–[4].

During 2010, the NAI organized two virtual scientific workshops, aptly named “Workshops Without Walls” (WWW) [5]–[7]. The overarching goal of these workshops and other astrobiology “all-access” events is to promote the exchange of knowledge between scientists of diverse disciplines by facilitating their interaction across distance. The basic philosophy is to allow participation from any device (e.g., videoconference system, laptop, mobile device; see Box 1) and location (e.g., videoconference room, office, home). These workshops, as with other NAI all-access events, are free of charge and open to all. For the case study presented here, social media and science blogs played a role in expanding the discussion to include various sectors of the public. The need for travel is eliminated, along with the associated environmental impacts, cost savings are significant, and—most importantly—science is made available to a much broader audience. Adhering strictly to the scheduled agenda allows participants to “tune in” for talks of particular interest, thus enabling them to interleave workshop participation with attending to other professional or personal matters.

**Box 1. Technical Implementation**

The technical philosophy for this workshop was to allow attendees to join from any device and any location. Some attendees joined the meeting using only a Web browser, some joined from a videoconferencing room, and some joined from mobile devices.

***Videoconferencing.*** Videoconferencing systems allow rooms of people to interact over distances using high quality audio and video. H.323 is the standard used by most videoconferencing systems available today. The most popular manufacturers of videoconferencing equipment are Polycom, Tandberg, and Lifesize. Most universities and government facilities have such systems available.

A typical videoconferencing system (or *endpoint*) is able to connect to one other system (site). Higher end systems may allow connecting up to four additional sites. When connecting to another system, the IP address of the remote system is used as the “phone number” for the remote endpoint. Older systems based on H.320 ISDN technology use an actual phone number instead of an IP address. If the need to mix IP and ISDN connections arises, additional equipment may be required. If a virtual meeting involves participation from multiple sites (i.e., physical locations), it is possible to connect many remote endpoints together using a videoconferencing bridge. A typical bridge can connect anywhere from 20 to 80 sites to a single videoconference, mixing H.323, H.320, and phone technologies.

Desktop H.323 videoconferencing software clients with acceptable fidelity have recently become available (examples include Dylogic Mirial and Tandberg Movi). These clients run on a computer or mobile device, and use a Webcam and microphone to communicate with other H.323 systems. This software can be used when a meeting participant is unable to join from a standard H.323 videoconferencing room. It is important to note that popular video chat programs such as Skype, iChat, Gtalk, and others are not compatible with H.323 videoconferencing.

***Online meeting software.*** Online meeting software such as Adobe Connect and WebEx allows users to participate in the meeting using only their computer. Several features are required to make this possible, which include:

**Screen sharing** enables participants to see what the presenter is showing (powerpoint slides, movies, etc.).

**Integrated video** makes it possible for participants to see the presenter. The presenter can also optionally see the participants.

**Audio (VoIP)** allows participants to hear the presenter and discussion.

**Chat** facilitates participant discussion and question and answer.

**Cross-platform support** gives participants the capability to join from the operating system/device of their choice.

**Recording** is allowed by most online meeting software tools.

***Teleconferencing.*** Teleconferencing can be integrated as needed, for example, when a presenter is unable to join from a videoconference facility. In that case the presentation can be made from a personal computer. An integrated or separate Webcam can provide video of the speaker, and a telephone connection can provide two-way audio.

***Resolving technical issues.*** For attendees joining meetings using online meeting software with integrated audio and video such as Adobe Connect, the most common technical issue was audio and/or video freezing. This issue is frequently resolved by exiting the meeting room and rejoining. Bandwidth issues are uncommon, as the meeting software will adjust the bandwidth used to match the connection speed of the attendee.

In general, the reliability and speed of networks at universities and government agencies is more than adequate to support H.323 videoconferencing. Best practices such as muting microphones when not speaking, zooming the camera in on the speaker, and muting audio from the meeting software to prevent feedback loops help to ensure a solid videoconferencing experience. Having a dedicated videoconferencing bridge operator monitor and equalize audio levels among connected sites improves the experience further. In all cases, testing the videoconferencing and meeting software connections for each speaker in advance is essential for avoiding problems and keeping the meeting focused on content rather than technical issues.

## Case Study

**Figure 1:**
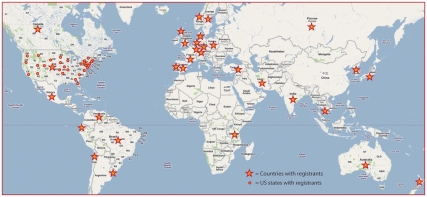
Over 560 people from 31 US states and 30 other countries registered for the November 2010 workshop. (Red dots represent the US states with WWW registrants; red stars represent the countries with WWW registrants) [7].

In November 2010, the NAI organized its second Workshop Without Walls on “Molecular Paleontology and Resurrection: Rewinding the Tape of Life” (Figure 1). Scientific organization was provided by researchers from the NAI teams at the Georgia Institute of Technology (Georgia Tech) and Montana State University. Technical support was provided by the NAI Central management organization at the NASA Ames Research Center.

**Figure 2:**
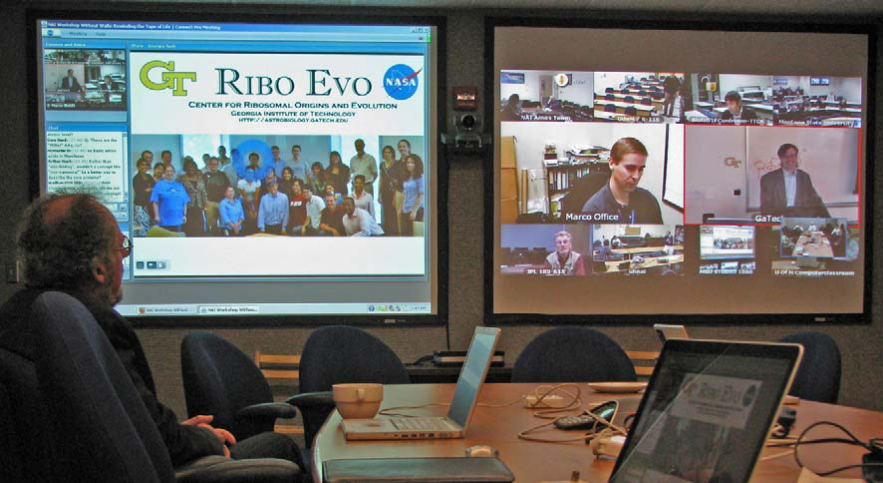
Dual screens in a videoconferencing room at the NASA Astrobiology Institute. On the left screen is the online meeting software showing speaker slides, video, and chat windows. On the right screen is a mosaic of other videoconferencing rooms connected for the workshop. Over the course of the November 2010 workshop connections were made to 21 different videoconferencing sites at US and international institutions. The video feeds were integrated with Adobe Connect and broadcast live to participants around the world, who could ask questions through an integrated chat window. In addition, presentations were recorded and have been archived and made available to the public via the internet [7].

The Workshop Without Walls concept was developed by the NAI as part of its mandate to use modern information technology to foster interdisciplinary and collaborative research among widely distributed investigators. Astrobiology is the study of “the origins, evolution, distribution, and future of life in the universe” [8],[9]. Astrobiology's breadth and interdisciplinarity requires collaboration among scientists with expertise in various fields. For example, it would not be unusual for experts in molecular biology, ecology, paleogenetics, geochemistry, biochemistry, and thermodynamics to work together on an astrobiology research project [10]. This collaborative research framework has international reach as well. The NAI has international partnerships with the Australian Centre for Astrobiology and the Centro de Astrobiología in Spain, among several others [11]. In accord with the goals of the NAI to foster international collaborative research, the workshop featured talks by scientists from 20 academic institutions around the world, including presentations streamed from the US, Denmark, Japan, and Canada (Figure 2).

The presentations were organized into six topical sessions held over 3 days. Presentations were recorded, including question and answer periods, and are available for viewing on the Web [7]. The presentations sought to address the prevailing approaches to origin of life research, which include understanding (1) which of the chemical components of modern life could have formed from abiotic processes (bottom-up approach) and (2) which of the constituents of modern life could have been a part of more ancestral and simpler forms of life (top-down approach).

The first day of the workshop focused primarily on the origin and early evolution of life, prior to 3.8 billion years ago (Ga). Talks included overviews of research directed at probing the role of iron-sulfur minerals that were likely present in early Earth environments and may have catalyzed reactions that supported early life (carbon monooxide condensation, dinitrogen fixation, etc.). These talks were complemented by presentations on prebiotic chemistry.

Day 2 of the workshop focused on research aimed at understanding the constraints imposed on the evolution of life during the dynamic early history of Earth (>2.0 Ga). The day opened with talks on linking major changes in the Earth's geosphere (e.g., accumulation of atmospheric O_2_) with changes recorded in the genetic or evolutionary record (e.g., origin of metabolisms that utilize O_2_). The second session on Day 2 focused primarily on the use of evolutionary theory to reconstruct extinct proteins and the resurrection of these proteins for experimental study. Day 3 opened with talks on the study of extant microbial diversity and physiology that provided context to the findings presented on Days 1 and 2. The workshop closed with presentations on the evolution of information (DNA and RNA) pathways.

The breadth of the six topical sessions promoted significant discussion among workshop participants. Discussions occurred both within the interactive workshop itself during the question and answer periods as well as in online chat rooms (see Box 1 for more details). Some of these discussions may lead to new interdisciplinary collaborations, one goal of the workshop. For example, a new idea emerged to apply ancestral state reconstructions to create “evolutionary intermediates” for use in probing the changing biochemical reactivities and substrate utilization patterns during the stepwise evolution of metalloproteins [12]–[14] as well as to understand how ancient environmental conditions, such as pH and temperature, affected the evolution of thioredoxin enzymes [15].

One of the participants, Jennifer Glass, in summarizing her experience, stressed, “The flexibility of having the option to come and go in between classes and lab work coupled with free registration and the exciting topic made the virtual workshop very attractive to our team members. For those who had previous obligations, the benefit of having all of the talks recorded and posted on the Website for future viewing was extremely helpful… The potential disadvantage of not being able to directly interact with others at the workshop was ameliorated by the chat box on the Adobe Connect window…. As an early career researcher, this workshop has inspired me to grow my research into new areas…”.

## Broadening the Reach: Benefits for Science Collaboration and Public Participation

Seventy-six percent of respondents to the postworkshop survey [7] said that they would not have traveled to the conference in person. This could have been because of scheduling conflicts, lack of travel funds, or, as some survey respondents indicated, the workshop was in a specialized area for which they could not justify the time and expense to attend in person but for which virtual attendance was highly cost effective. Thus, the virtual nature of the WWW enabled the participation of a larger and more diverse group of scientists—promoting greater understanding and collaboration around the globe. In response to the survey question “Did you find opportunities for collaboration?” 38 individuals (55% of those who answered the question) indicated that they did or might find opportunities for collaboration as a result of the workshop [7].

In addition, many nonscientists, who were highly unlikely to attend an in-person scientific meeting, were able to participate. These included educators, science writers, and people who simply have an interest in astrobiology or perhaps just wanted to see a “real” science conference in action. The speakers addressed the public audience by including brief introductory materials in their presentations to put the science in context for a lay audience. Several of the participants embraced the opportunity to experience undiluted science. One wrote, “This is great! I'm not in the educational world, but as a passionate amateur, I'm really grateful that you've given me the opportunity to listen in on this. Even if I only understand a tenth of it, that's a bunch of learning I would never have had the opportunity to do! Thanks!” Another wrote, “…I had to teach during most of the conference… I did wow my 7th graders with a couple of minutes of a live science conference for the fun of it.” Public attendance at science conferences is rare—in part because of high conference registration fees and travel costs. The free, open access WWW format is breaking new ground in encouraging and enabling public exposure to science.

## Economic Impact and Ecological Benefits

### Mitigating the Carbon Footprint

In a recent letter to *Science* “Travel Trade-Offs for Scientists,” I.C. Burke suggested that scientists need to lead the way in reducing carbon emissions due to conference travel [16]. In agreement with this progressive concept, the virtual Workshop Without Walls enabled the broad participation of ∼560 registrants with no travel required. Of the 140 people who filled out the postworkshop survey, 34 (∼24%) indicated that they would have traveled to the workshop in person. Of these, 26 were from the non-Atlanta area for which travel cost and environmental impact estimates were made. We estimate that ∼290,000 km would have been traveled by these participants had they attended the workshop physically. This is nearly the distance between the Earth and the moon; a distance that would have left an estimated carbon footprint of ∼26 tonnes if traveled by air. This corresponds to ∼1 tonne per attendee, which is more than the average CO_2_ emission per capita per year for ∼one-quarter of all world nations (US average is 18.9 tonnes per person per year) [17].

### Mitigating Traditional Travel Costs

Conventional meetings require a large financial budget to support the physical travel of attendees, including airline travel, venue fees, lodging, and food expenses. For the 26 individuals who indicated interest in attending this conference in person, it is estimated that roughly US$20,000 in airline travel was saved as a result of this workshop being hosted virtually (see Text S1). Using the US federal per diem rate for domestic travel, an estimated US$24,000 would have been spent on food or lodging for these 26 attendees. Thus, in total, roughly US$44,000 was saved, corresponding to ∼US$1,800 per attendee. Importantly, this is conceivably a low estimate of cost savings, since roughly 60% of the workshop participants did not complete the postworkshop survey, from which these estimates were derived. Likewise, venue fees and other costs were not factored into this overall estimated cost savings.

## Concluding Remarks

A central goal of the NAI and the NASA Astrobiology Program as a whole is to bring scientists from varying disciplines together to develop new ideas and forge new interdisciplinary research collaborations. This has traditionally been accomplished through in-person meetings. The WWW concept fosters these same goals with even broader participation by eliminating the requirement for physical travel.

Besides being an efficient way to promote novel collaborations, Workshops Without Walls and other astrobiology all-access events bring “science to society” by opening the doors of scientific meetings to the public and allowing all attendees (both scientists and lay-public) to ask questions during the meeting. The Workshop Without Walls format also provides an opportunity for students—who might not otherwise be able to attend—to experience the field of astrobiology, and may encourage their interest at an early stage in their careers.

These virtual workshops will continue to improve in the future. The NAI is experimenting with “virtual hallway” chat areas to facilitate additional interactions between the scientists and with the public. Integrating the use of social media (such as Facebook and science blogs) is another way to extend the workshops' public reach. Areas in which there is a need for increased public understanding of science (such as climate change and evolution) may thus be particularly appropriate topics for future Workshops Without Walls.

## Supporting Information

Text S1 **Methods.**
(DOCX)

## Abbreviations

NASA: National Aeronautics and Space Administration
NAI: NASA Astrobiology Institute
WWW: Workshop Without Walls
