# Supplementary material for: Workshops without Walls: Broadening Access to Science around the World
Source: PLoS Biol. 2011 Aug 2;9(8):e1001118. doi: 10.1371/journal.pbio.1001118 (PMC3149038; doi:10.1371/journal.pbio.1001118)
Supplement: Text S1 — Methods. (DOCX) [file pbio.1001118.s001.docx]

**Methods**

If this had been an in-person meeting, we assume that it would have been held in Atlanta, Georgia (hosted by the NAI’s Georgia Tech team). Therefore any participants who are from the Atlanta area were not included in calculations of travel costs. The virtual workshop was held on workshop on January 19-21^st^, with January 18^th^ and 22^nd^ considered as travel days.

Of the 560 total registrants, ~ 140 responded to the post-workshop survey, which included the question of whether they would have physically attended the workshop if the virtual internet-based platform was not available. Of the 140 attendees who answered this question, 24% (36 individuals) indicated that they would have traveled to attend the workshop. Of these, 26 individuals were from the non-Atlanta area and provided the physical location from which they participated in the virtual workshop. This physical location was used to estimate their travel costs and carbon footprint had they attended the workshop in person.

Travelocity (http://www. travelocity.com) was used to identify the cheapest flight for a given registrant to the Atlanta, Georgia airport. The closest airport for departure for each attendee was determined using the airport finder application at http://www.travelmath.com/closest-airport. Airline-specific carbon estimates were made for each leg of the trip using an application at http://www.terrapass.com/carbon-footprint-calculator/. This calculator compensates for the type of aircraft that is used for the flight, how much fuel it uses, and how many people (number of travelers) this fuel load can be spread across.

For all participants, 2010 United States government per diem rates were assumed for the Atlanta area (http://www.gsa.gov/portal/category/21287), which amounted to $188 per day ($132 per day for allowable lodging expenses and $56 per day for allowable meals and incidental expenditures). This value was multiplied by the total number of travel days (5 days) resulting in a total of $940 per participant. Although many participants are from other countries, we assume similar per diem rates for these individuals.
